# Simian Foamy Virus Co-Infections

**DOI:** 10.3390/v11100902

**Published:** 2019-09-27

**Authors:** Shannon M. Murray, Maxine L. Linial

**Affiliations:** Division of Basic Sciences, Fred Hutchinson Cancer Research Center, 1100 Fairview Ave N, Seattle, WA 98109, USA; mlinial@fredhutch.org

**Keywords:** foamy virus, spumaretrovirus, co-infections, NHP, pathogenesis, zoonoses

## Abstract

Foamy viruses (FVs), also known as spumaretroviruses, are complex retroviruses that are seemingly nonpathogenic in natural hosts. In natural hosts, which include felines, bovines, and nonhuman primates (NHPs), a large percentage of adults are infected with FVs. For this reason, the effect of FVs on infections with other viruses (co-infections) cannot be easily studied in natural populations. Most of what is known about interactions between FVs and other viruses is based on studies of NHPs in artificial settings such as research facilities. In these settings, there is some indication that FVs can exacerbate infections with lentiviruses such as simian immunodeficiency virus (SIV). Nonhuman primate (NHP) simian FVs (SFVs) have been shown to infect people without any apparent pathogenicity. Humans zoonotically infected with simian foamy virus (SFV) are often co-infected with other viruses. Thus, it is important to know whether SFV co-infections affect human disease.

## 1. Introduction

Foamy viruses (FVs) comprise the *Spumaretrovirinae* subfamily of the family *Retroviridae* and are also designated spumaretroviruses [1]. FVs are ancient complex retroviruses that have co-evolved with their nonhuman primate (NHP) hosts for at least 60 million years [2]. Interestingly, recent sequencing of an ancient marine fish, the coelacanth, revealed an endogenous foamy virus (FV) [3]. The coelacanth is an ancient marine four-lobed fish believed to be the organism that first became terrestrial and is the ancestor of all terrestrial organisms. This indicates that FVs have existed for an estimated 400 million years [3], making *Spumaretrovirinae* the oldest known extant vertebrate virus subfamily. 

FVs are apparently nonpathogenic in their natural hosts, which include NHPs (reviewed in [4]), felines [5], bovines [6], and equines [7]. FVs have also been found in bats, although the physiological consequences were not stated [8]. In non-primate hosts, FV prevalence is reported to be between ca. 30–70% in adults, depending on age, host, and location (reviewed in [9]). FVs were first identified by their cytopathicity in tissue culture cells ([10,11,12], reviewed in [13]). Thus, there have been many efforts to determine whether these viruses are pathogenic in vivo. To date, there have been no reports demonstrating clear-cut pathogenicity in natural or accidental hosts (reviewed in [14]). However, in research settings, there is some evidence that FVs can exacerbate the pathogenesis of other viruses. This phenomenon cannot be well studied in natural infections, as finding FV uninfected adult animals is difficult. 

Simian FVs (SFVs) replicate primarily in tissues of the oral mucosa [15,16], and transmission most likely occurs through transfer of saliva from one individual to the next. Often, infected saliva transfer occurs through grooming, biting, or sharing food (reviewed in [4]). It is also thought that SFVs from saliva enters the blood or oral cavity of the recipient [17]. In NHP blood transfusion studies, SFV can be transmitted through blood [17,18]. However, it is not known whether this occurs in natural settings. In natural FV-infected felines, FV DNA has been detected in buccal swabs and in the blood [19].

While humans have contacts with felines and bovines, there is no evidence for actual infection of humans with FVs from these species (reviewed in [14]). Zoonotic infections with SFVs are frequent among animal caretakers, zoo keepers, bushmeat hunters, and others in direct contact with NHPs [20,21,22,23]. For example, ca. 2–5% of individuals in North America who report contact with NHPs are FV-infected, as determined by SFV polymerase chain reaction (PCR) (reviewed in [14]). There is no evidence for human-to-human transmission. The underlying reason(s) as to why the virus has not adapted to humans, but to all other primate species, is unknown.

Interestingly, SFV replication takes place in the most terminally differentiated superficial epithelial cells of the oral mucosa—those about to slough off into saliva [15]. The lack of pathogenicity of FV infections may be a result of this replicative niche in a cell type that turns over rapidly and is relatively dispensable to the host [15]. If this cellular niche for SFV replication is altered as described below for experimental simian immunodeficiency virus (SIV) infections, there may be potential for pathogenic effects.

## 2. FVs and the Virome 

Each organism is host to a multitude of microbes, known as the microbiome. Organisms are also host to a collection of viruses, known as the virome (reviewed in [24]). Since, in natural species, FVs infect the majority of adults, FVs are considered part of the virome. It is well established that the virome plays a role in health and disease and that co-infecting viruses can affect each other and the microbiome [24]. In this review, we will consider the effect of FVs on infections by other viruses, in some cases, pathogens, and we will also consider the effects of other viruses upon FV infections, summarized in Table 1. 

## 3. SFV Zoonotic Infections 

There is evidence for zoonotic transmission of SFVs to humans (reviewed in [14]). Many of the zoonotically infected people are those with direct contact with NHPs, including bushmeat hunters in Africa as well as zoo and laboratory workers [20,21,22,23,29]. People whose interactions with NHPs are less direct but who are SFV-infected have been found in Asia [30]. There are small numbers of individuals identified who are infected with both SFV and human immunodeficiency virus (HIV) [31,32]. Moreover, as there is interest in using FV as a vector for gene therapy [33,34], the questions surrounding FV co-infections are becoming more relevant.

## 4. SFV and Other Retrovirus Co-Infections

SFVs are endemic in all NHP species examined to date in Africa, Asia, and the Americas [13,35]. In Africa, there are at least three other endemic complex retroviruses that infect NHPs. These are SIV, simian T-cell lymphotropic virus-1 (STLV-1), and simian retrovirus type D (SRV D). SIV had been thought to be nonpathogenic in its natural African monkey hosts [36], but there are reports of an acquired immunodeficiency syndrome (AIDS)-like illness in some SIV-infected natural hosts [37,38]. Additionally, when SIV infects other NHP species, such as chimpanzees and gorillas, it can be pathogenic in some cases [39,40,41].

STLV-1 has not been as extensively studied in its natural NHP hosts as human T-cell lymphotropic virus-1 (HTLV-1) has been in humans. HTLV-1 is pathogenic in only a small fraction of infected humans (reviewed in [42]), and STLV-1 infection in NHPs has been associated with a number of T cell abnormalities, including lymphomas and leukemias (reviewed in [43]). Many Asian NHPs are infected with another retrovirus, SRV D [44,45]. Thus, it is likely that many macaques are co-infected with SRV D and SFV. In NHPs, SFV as well as cytomegalovirus (CMV), a herpesvirus, are endemic and therefore many adult animals are co-infected with both viruses [46]. However, whether these CMV or SRV D co-infections alter animal health has not been reported.

### 4.1. SFV and SIV Co-Infections

Researchers have used the rhesus macaque (RM), also known as *Macaca mulatta*, as a model for HIV infections. In the wild, SIV is not known to exist in Asia [47,48], the natural habitat of RM. SIV sooty mangabey (SIVsm) has been found to be pathogenic in RM [49] and has become the virus used to infect RM to recapitulate HIV infections. SFV infection is latent in most tissues, including the blood. SFV replication is only detected in oropharyngeal tissues [16,25]. A key question concerning FV–host interactions is whether FV replication is controlled by the host immune system. To address this question, RM infected with pathogenic SIV strains (SIVmac239 and SIVmac155T3) that lead to CD4+ T cell depletion were studied, and SFV replication was assessed in the blood and other tissues [25]. In SIV/SFV co-infected RM, SFV replication was expanded to include the small intestine, the jejunum [25], which is a site of SIV-induced CD4+ T cell depletion [50]. However, other tissues that were CD4+ T cell depleted were not permissive for SFV replication [25]. Thus, it is not only CD4+ T cell depletion per se that is responsible for the expansion of SFV replication to the jejunum. Overall, this indicates that the host immune system is not limiting systemic SFV replication. It is important to note that the strains of SIV used in this study were lab-adapted, highly pathogenic strains that are HIV infection models in NHPs and are unlike SIV strains in natural hosts, which are usually poorly pathogenic.

It is also possible that SFV can affect the pathogenicity of other viruses, such as SIV. In natural settings, most adult NHPs are naturally infected with SFV. Therefore, this issue was addressed using RM individually housed in primate center facilities. The researchers examined SFV− and SFV+ RM infected with a pathogenic SIV strain (SIVmac239) [26]. The SFV+ animals had increased SIV viral loads in the blood and died more rapidly than SFV− RM. Thus, SFV infection does exacerbate SIV pathogenesis in experimentally SIV-infected RM. 

The mechanism by which SFV increases SIV pathogenesis is unknown. One tissue target of SIV pathogenesis in RM is the jejunum [50]. Whether SFV replication in the jejunum contributes to this pathogenicity is an outstanding question. Because the SIV RM model commonly uses SFV+ animals, the RM model might not totally recapitulate HIV pathogenesis in people who are SFV−. There is evidence of HIV/SFV co-infections in humans [29,32]. Bushmeat hunters often get bitten by NHPs and become SFV-infected [29]. The number of bushmeat hunters co-infected with SFV and HIV is small. It would be of interest to compare HIV infections of bushmeat hunters who are SFV-infected or uninfected.

Free-living chimpanzees (cpz) in multiple countries of Africa, such as Cameroon and Gabon, have been analyzed for infection with SFVcpz and SIVcpz [51]. The researchers found that 15/70 (~21%) of SFV-infected animals were co-infected with SIV. This indicates that co-infection of these two viruses is common in chimpanzees. In another study, in the monkey the Ugandan red colobus [52], SFV/SIV co-infections were also common, with 23% of individuals co-infected. The routes of transmission of these two viruses are apparently different, with SFV transmission primarily through saliva. In the case of SIV, both sexual transmission and aggressive behavior have been implicated (reviewed in [53]). The effects of these two viruses on the health of free-living NHPs was not noted in either study, but this would be of great interest because these are clear examples of natural co-infections. 

### 4.2. SFV and STLV-1 Co-Infections 

A cohort of naturally SFV-infected baboons were studied. Some of the baboons were also naturally infected with STLV-1, while others were not, with 18 baboons studied per group. It was found that SFV DNA levels in the blood, but not in the saliva, were increased in the STLV-1-infected baboons [27]. It is not known why there is increased SFV DNA in the blood after STLV-1 infection. Two possibilities are (1) increased integrated SFV proviruses, or (2) increased numbers of SFV viral particles in the blood. The authors show that, in tissue culture cells, the related HTLV-1 transcriptional activator protein (Tax) can stimulate the FV long terminal repeat (LTR). They suggest that this could be a mechanism that explains the in vivo results [27]. However, it is also possible that since STLV-1 increases the number of lymphoid cells in the blood, this could result in more SFV in the blood as well. Overall, STLV-1 infection is unlikely to alter SFV transmission, since SFV transmission is mostly through saliva rather than blood. It is not known whether SFV infection alters the pathogenicity of STLV-1, since all of the STLV-1-infected animals were also SFV-infected. There is also concern about SFV/HTLV-1 co-infection in humans [54], as discussed further below.

### 4.3. SFV and SRV D Co-Infections 

SRV D infections in Asian primates such as macaques sometimes causes an immunodeficiency-like syndrome [44,45]. SRV D appears to be transmitted through saliva as well as urine and feces [55]. There is a report of one worker who is seropositive for both SFV and SRV D [56]. It should be noted that this individual was both seropositive and PCR positive for SFV, but could not be shown to be PCR positive for SRV D. Thus, this person may have been exposed to both viruses, producing antibodies, but may not be actively SRV D infected. There is no evidence that this SRV D/SFV seropositive human has any retroviral-related pathogenesis.

## 5. SFV Zoonotic Co-Infections and Hematological Changes

One study identified HTLV-1 and SFV co-infected hunters in Central Africa [54]. Fifty-six percent of the hunters bitten by NHPs and infected with HTLV-1 were also infected with SFV, suggesting that these two viruses may have been co-transmitted via the bites [54]. However, the pathogenic effects of the co-infection were not evaluated. 

There is a publication presenting evidence that SFV-infected bushmeat hunters from Cameron seem to have different levels of hematological markers, including hemoglobin, creatine phosphokinase, and bilirubin, than SFV-uninfected bushmeat hunters [57]. The SFV-infected group had a higher incidence of HTLV-1 infections, and all participants except for one were infected with hepatitis B virus (HBV). Thus, it is hard to ascribe the differences in hematological markers to SFV infection alone. These differences could result from SFV co-infection with either HTLV-1 or HBV. In fact, as described in baboons infected with STLV-1 [27], FV DNA levels were increased in the blood of co-infected animals. Another publication reports no hematological differences in SFV-infected North American research and zoo workers occupationally exposed to NHPs [58]. Since these individuals were unlikely to be infected with HTLV-1 and/or HBV, this supports the notion that hematological changes from SFV infection are a result of co-infections. In natural hosts, there is no evidence that SFV alone leads to any hematological changes. However, hematological changes in natural hosts infected with FV have not been thoroughly investigated.

## 6. Non-Primate FV Co-Infections

Most work on FV transmission has been done in NHPs. This has served as the general model for FV transmission in other natural species. While the transmission route for non-primate FVs has been speculated to include saliva, other transmission modes seem to occur. For example, bovine foamy virus (BFV) has been detected in bovine breast milk [59]. In fact, BFV has been shown to be transmitted from mothers’ milk to calves [60].

### 6.1. BFV and Herpesvirus Co-Infections

A serious illness of unknown origin in dairy cows is non-responsive post-partum metritis (NPPM). It is thought to be caused by a virus, as it is unresponsive to antibiotics. There is some evidence that bovine gammaherpesviruses (BoHVs) are associated with this disease (M. Materniak-Kornas, personal communication, 2019). Since many cows are infected by BFV [60], it is possible that co-infection of BFV and BoHV-4 or BoHV-6 is a factor in the disease. However, the researchers found that is not the case, since as many healthy cows as sick cows were infected with BFV (M. Materniak-Kornas, personal communication, 2019). 

### 6.2. FFV and Other Retrovirus Co-Infections in Cats

Feline leukemia virus (FeLV), a retrovirus of cats, often leads to fatal diseases, including lymphomas [61]. One study examined FeLV and FFV co-infections in domestic cats [28]. Natural FeLV infections can have two outcomes; these are progressive infections leading to disease, or regressive infections. Regressive infections are characterized by a transient production of viral structural proteins and a persistence of low levels of integrated viral DNA. Cats that are progressively infected have detectable FeLV RNA and infectious virions. It was found that higher levels of FFV DNA in the blood correlated with increased FeLV viremia and disease progression [28]. In another feline study, the researchers examined FeLV regressive versus progressive infections [19]. In cats with regressive infections, there were lower levels of FFV DNA in the oral cavity. This suggests the possibility that innate or adaptive immunity to FeLV in the regressors could affect FFV as well. 

There are cats co-infected with FFV and feline immunodeficiency virus (FIV), a lentivirus causing decreases in CD4+ T cells and feline acquired immune deficiency syndrome (FAIDS) [62]. In one study, a small number of cats were experimentally infected with FIV alone or with FIV and FFV. In this study, FFV co-infection did not enhance the pathogenesis of FIV [63]. This is in contrast with what was seen in RM infected with SFV and SIV, as described above [26]. However, in a study of FFV experimental infection, FFV RNA was rarely seen in saliva samples (in only 1 of 80 samples) [64]. Thus, the FIV/FFV co-infection study might be flawed, since it is possible that FFV experimental infection does not mirror natural FFV infections. More studies are needed to assay viral RNA in tissues of naturally FV-infected non-primates, including cats. 

## 7. Conclusions

In natural hosts, FVs are highly prevalent and therefore finding uninfected adult animals is difficult. Thus, all information about the effect of FVs on other viral infections in these species involves artificial situations in which some animals are kept FV-free. Most of the studies on FV effects on other viral infections use lab-adapted pathogenic viruses. In research laboratory settings, there is clear indication that FV infections exacerbate lentiviral outcomes. There is also evidence that SFV has an expanded tissue tropism in lentiviral co-infected NHPs. The prevalence of FV co-infected individuals has been described in natural hosts. However, these studies did not report whether FV co-infection affects pathogenesis induced by other viruses. Therefore, it is not known whether the results from lab settings are relevant to what occurs in natural settings. The mechanism of how FV infections may affect other viral infections is not clear. 

Humans can be zoonotically infected with SFVs from NHPs. While it has been reported that there are people co-infected with SFV and HIV or HTLV-1, there are almost no data about whether SFV infection of humans can exacerbate other viral infections. Overall, more studies in natural settings and of human zoonotic FV co-infections would be important to understand whether FVs contribute to the pathogenicity of other microorganisms. One example of a FV pathogenic effect on hosts is in RM, using lab-adapted strains of a lentivirus, SIV. In the future, it will be important to see whether HIV pathogenesis is worse in humans co-infected with SFV. It is also possible that in humans, FV infections could exacerbate other viral infections such as HTLV-1, HBV, or even CMV or other herpesviruses. The understanding of how SFVs affect other human viruses is important, given the potential use of FV vectors for gene therapy to treat human diseases.

## Figures and Tables

**Table 1:** Foamy virus (FV) and retrovirus co-infections: their effects on the host.

Co-Infecting Retrovirus	Genus	Host Species	Effects on FV	Effects of FV	References
Simian Immunodeficiency Virus (SIV)	*Lentivirus*	*Macaca mulatta* (rhesus macaque)	FV replication expanded to the jejunum	Increased SIV RNA loads in the blood	[25]
				Decreased survival and greater CD4+ T cell loss	[26]
Simian T cell-Lymphotropic Virus (STLV-1)	*Deltaretrovirus*	*Papio anubis* (baboon)	FV proviral load increased in the peripheral blood, but not in saliva	Not determined	[27]
Feline Leukemia Virus (FeLV)	*Gammaretrovirus*	*Felis catus* (domestic cat)	Higher FFV DNA loads in PBMC correlated with increased FeLV viremia and disease progression	Not determined	[28]
			FFV DNA in the oral cavity was detected in more cats with progressive FeLV disease compared to regressive FeLV disease		[19]
